# Socioeconomic determinants of COVID-19 incidence in a German rural area

**DOI:** 10.1186/s12889-026-27285-5

**Published:** 2026-04-10

**Authors:** Konstantin Naoki Fujiwara, Ernst-Dieter Lichtenberg, Rejane Golbach, Fabian Holzgreve, Eileen M. Wanke, David A. Groneberg, Eva Herrmann, Daniela Ohlendorf

**Affiliations:** 1 Institute of Occupational, Social and Environmental Medicine, Goethe University Frankfurt, Frankfurt, Germany; 2 Bad Kreuznach Health Office, Bad Kreuznach, Germany; 3 Institute for Biostatistics and Mathematical Modeling, Goethe University Frankfurt, Frankfurt, Germany

**Keywords:** SARS-CoV2, Covid-19, Socioeconomic factors, Rural, Local level

## Abstract

**Introduction:**

This study examines the relationship between local-level socioeconomic factors and the incidence of COVID-19 infections in rural Germany. It does so by analyzing small spatial units (i.e. municipalities in the district of Bad Kreuznach, Rhineland-Palatinate), thereby addressing the research gap in the context of rural areas. By examining localized dynamics, the study sheds light on the socioeconomic determinants.

**Methods:**

An anonymized dataset of all reported COVID-19 cases of unvaccinated persons with confirmed SARS-CoV-2 infection in the rural district of Bad Kreuznach between 03/04/2020 and 01/06/2021 was analyzed using a negative binomial generalized linear model. The following socioeconomic factors were considered: GISD employment subscore, income tax per resident (at municipality and municipal association levels), population density, number of residents, presence of a hospital, and regional population potential.

**Results:**

A statistically significant influence of the variable income tax per capita was found at both the municipal level (coefficient − 1.072; *p* = 0.0224) and at the municipal association level (coefficient 2.914; *p* = 0.0033). A significant correlation was also found with the presence of a hospital (coefficient 0.773; *p* = 0.0091). Excluding the city of Bad Kreuznach did not change the main results.

**Discussion:**

In rural areas, the influence of socioeconomic factors was less pronounced than in studies of metropolitan regions and larger spatial units. In this study, only income tax per capita and the presence of a hospital were statistically significant, which may have been affected by unobserved confounders. Further research on smaller spatial units is needed to better understand the local impacts of socioeconomic factors and support targeted public health policy decisions.

## Introduction

The emergence of the coronavirus (SARS-CoV-2) in Wuhan, China, in January 2020 initiated a pandemic that has persisted for over two years, infecting more than 650 million people and resulting in over 6.8 million deaths worldwide [1, 2]. Early research identified several individual risk factors for severe COVID-19 progression, including advanced age [3, 4], obesity [3, 5], cardiovascular disease [6–8], and chronic conditions such as lung disease [3, 6, 7, 9], liver disease [3, 7, 10], and kidney disease [3, 5, 7], dementia [10], diabetes mellitus [3, 5, 7], and cancer [10].

In addition, the COVID-19 pandemic has exposed health inequalities, with socioeconomic factors playing a critical role in shaping infection rates and outcomes [11]. While numerous studies have examined these dynamics in urban [12–14] and regional [15–18] contexts, rural areas, which are home to a significant proportion of the population, are underrepresented in this discourse [19, 20]. Studies that included rural areas did so by focusing on large spatial scales (comparing a large number of districts rather than examining one district), such as nationwide studies in the US [21, 22]. These studies demonstrated significant disparities in SARS-CoV-2 incidence and mortality linked to socioeconomic factors such as income inequality, unemployment, housing conditions, and population density [21, 22]. Similar effects have also been documented in Japan [17], Brazil [18], South Africa [15] as well as individual states, regions and cities, such as Barcelona [13], Geneva [14] and New York [12].

Findings on the nationwide influence of socioeconomic factors in Germany have already been collected (23). It was shown that, in the first phase of the pandemic, residential areas with a higher socioeconomic status initially had higher incidences, which could be primarily explained by the return of holidaymakers from the Alps and business travelers [23, 24]. However, as the pandemic progressed, this trend reversed, with the deprived and socially weaker regions accounting for a larger proportion of SARS-CoV-2 infections [23, 24]. These studies [23, 24] included socioeconomic factors such as average income, university education, unemployment rate, employment rate, and GDP. Similar associations between socioeconomic factors (e.g. migration background, unemployment, rent index, deprivation indices) and incidence of SARS-CoV-2 were found in regional German studies focusing on states such as Bavaria [25] and cities such as Cologne [26, 27]. However, much of this research has focused on urban areas or broad regional datasets, leaving rural contexts underexplored.

Structural differences have already been highlighted between rural and urban settings, which are reflected in various areas, including economic opportunities, access to healthcare, and social capital [28–30]. These disparities can affect the quality of life, social interactions and educational and professional prospects of the respective residents.

Theories such as the “dual structure theory of urban and rural areas” propose that there are fundamental differences in the economic and social structures of urban and rural areas [31]. Urban and rural areas are distinct yet interdependent systems that coexist [32]. Contrary developments in urban and rural areas, such as the decline of rural areas and the evolving prosperity of urban areas, have been widely debated [29, 33]. There are also differences in social capital and the functioning of social networks between urban and rural areas. Sorensen et al. [30] demonstrated that rural areas have significantly higher bonding social capital, while bridging social capital is more prevalent in urban areas. Furthermore, rural residents were found to have a poorer health status, yet make less use of primary and specialist healthcare services, even after adjusting for age, race, ethnicity, gender, smoking, and health status [28]. Also, rural residents had less access to health information from various sources, such as primary care providers, specialist doctors, blogs, magazines, and search engines, than urban residents [34]. Economic growth patterns also differ between urban and rural areas, with urban areas generally experiencing higher growth rates [35]. This can be partly explained by the differing industry compositions in rural and urban areas [35].

Studies have highlighted significant disparities in healthcare access, social capital, and economic opportunities between urban and rural areas in Germany [36–38]. These disparities should be addressed in the context of “place or space effects on health” as well, considering the geographical dimension of population health [39].

Based on these findings and the association between socioeconomic factors and the incidence of COVID-19, the present study retrospectively examines whether socioeconomic factors influence the incidence of COVID-19 differently in rural municipalities compared to urban settings in Germany. The study focuses on small spatial units to capture localized dynamics that are often overlooked in broader analyses. Although rural areas are home to nearly 60% of Germany’s population and the majority of the European Union’s population [40, 41], their socioeconomic and epidemiological dynamics remain insufficiently studied. While prior research has analyzed socioeconomic factors at regional [25] or nationwide levels [23, 24], this study uses municipalities as small spatial units to reveal localized patterns that may be obscured in larger datasets. These analyses will be based on the example of the district of Bad Kreuznach, a predominantly rural area in Germany. The district was selected as a case study due to its level of rurality, its combination of small towns and surrounding villages with no direct link to a metropolitan area, and its economic characteristics, which are consistent with those of other rural districts. This is in contrast to some of the more economically strong districts in Bavaria and Baden-Württemberg. The heterogeneity of rural areas, combined with their socioeconomic and infrastructural characteristics, requires a closer examination at smaller spatial scales. This study provides one of the first empirical assessments of these factors at the municipality level in rural Germany by analyzing socioeconomic indicators such as employment, income, and population density. Unemployment and income tax per inhabitant reflect economic circumstances [23, 42], with lower socioeconomic status often being associated with a higher risk of infection due to limited preventive options and unfavorable living conditions. Population density was used as a proxy for housing conditions, as denser areas may facilitate transmission through closer contact and limited outdoor spaces [43]. Regional Population Potential, representing accessibility and spatial interaction, was included to capture mobility and social mixing, both of which influence infection rates. While the variables provide meaningful insights, data constraints limited the inclusion of key factors such as educational attainment and employment sector distribution. The absence of these dimensions restricts the ability to explore mechanisms, such as the elevated risks faced by retail, hospitality, and healthcare workers.

This study aims to investigate the association between widely available socioeconomic factors and the incidence of SARS-CoV-2 in a rural context of Bad Kreuznach. The study does not aim to provide a comprehensive explanation of all mechanisms. Instead, the focus is on improving our understanding of how socioeconomic indicators relate to infection rates in rural small spatial units in Germany. Ultimately, this paper seeks to bridge a critical gap in the literature by demonstrating the importance of “thinking small” when informing health policies that address the needs of rural populations. By focusing on small spatial units, the study provides one of the first empirical analyses of socioeconomic determinants of SARS-CoV-2 incidence at the municipality level in rural Germany. It challenges urban-centric assumptions by examining rural contexts, and it establishes a connection between scientific research and policymakers, providing insights for public health planning and pandemic preparedness worldwide. Unlike comparable studies, which often examine metropolitan regions [12–14] or larger spatial units such as federal states [15–18] or entire countries [21, 22], this study focuses on a rural area with a homogeneous population structure on a much smaller spatial scale, examining individual municipalities. Therefore, the main difference between this study and others on the same topic is the geographical focus on rural areas and the significantly lower administrative level with smaller spatial units. Hence, the study addresses three research questions:

1. How do socioeconomic factors influence the COVID-19 incidence in rural municipalities?
2. In what way do these relationships differ from those observed in urban and regional studies?
3. What implications do these findings have for public health policies tailored to rural areas?

## Methods

This analysis used data from three different domains: baseline data on individual municipalities, available district data on selected socioeconomic influencing variables, and data on all reported COVID-19 infections in the Bad Kreuznach district between 03/04/2020 and 01/06/2021. The first infection with COVID-19 in this district occurred on 4 March 2020 and was officially confirmed on 10 March 2020 [44], after which several schools in the area were closed [45, 46]. From 16 March 2020, schools and daycare centers were closed throughout the state of Rhineland-Palatinate, followed the next day by further restrictions, including the banning of all events and the closure of retail units [47].

The research period was selected because it represents the spread of COVID-19 in the county before the introduction of COVID-19 vaccines. The first vaccination in the federal state of Rhineland-Palatinate took place on 27 December 2020. Initially, vaccinations were administered exclusively to high-risk groups, such as residents of nursing homes and medical personnel. Beginning on 7 January 2021, the vaccination campaign was expanded to include the general population.

There was an approved ethics application from the Rhineland-Palatinate State Medical Association (Number: 2023-17008-retrospektiv).

### Bad Kreuznach - exemplary analysed rural municipality

The district of Bad Kreuznach is centrally located in the federal state of Rhineland-Palatinate with a total population of 158,746 inhabitants across 118 municipalities [48].

It is divided into five “Gemeindeverbände” – henceforth translated to “collective municipalities” - (Langenlonsheim-Stromberg, Nahe-Glan, Kirner Land, Bad Kreuznach and Rüdesheim) and the unincorporated city of Bad Kreuznach [49]. A collective municipality is a territorial authority consisting of an association of several neighboring local municipalities that takes over administrative business and tasks at the municipal level [50]. The important economic sectors are agriculture, mainly viticulture and forestry, while the city of Bad Kreuznach is the largest industrial location [51]. Additionally, the district of Bad Kreuznach achieves an index value of 0.6 for rurality (very rural, good socioeconomic situation) in the “Thünen Landatlas”, making it an appropriate example of a rural district in the Federal Republic of Germany [52]. The degree of rurality is measured using the following criteria: built-up area, proportion of agricultural and forestry land, and location within the region [52]. The Thünen-Landatlas is provided by the Federal Ministry of Food and Agriculture (Bundesministerium für Ernährung und Landwirtschaft, BMEL) and is an interactive spatial database providing indicators that describe the demographic, economic, and structural characteristics of rural and urban regions in Germany. It is used to classify regional rurality and to analyze spatial disparities [52]. 

### Data collection of Covid-19 infections

Between 03/04/2020 and 01/06/2021, the public health department of the district Bad Kreuznach collected 2,921 case files on COVID-19 infections. The data originated from the department´s internal documentation, which was carried out using the “mikado” software program of the Mikroprojekt GmbH [53]. All cases reported by name with a positive PCR test result were recorded in accordance with compulsory registration [54], .

From the documentation in “mikado,” the following data points were used:

- Gender (male/female).
- Date of birth.
- Residential address.
- Date of positive PCR.

Following the exclusion of erroneous or incomplete data, 2,881 cases were included in the analysis.

As the data set was anonymized, it could only be assigned to the respective case files via the case number (the so-called PKNR). Age was determined using the date of birth, while the residential address was only used to infer the associated municipality. The data was accessed for research purposes on 27.04.2021.

### Socioeconomic factors

The following socioeconomic influencing variables were included: GISD employment subscore, income tax per municipality (€ per inhabitant), population density (inhabitants per km^2^), number of residents, presence of a hospital, and regional population potential (within 100 km in 1000).

Unemployment: The data for the GISD employment subscore was taken from freely accessible GISD data. The subscore is composed of the unemployment rate (municipal level), the employment rate (municipal level), and gross wages (county level) [55].

Income tax per municipality: The data on income tax was taken from the Rhineland-Palatinate Statistical Office. Under “My village, my town,” various data can be viewed at the municipal level [56]. Here, the income tax revenue is given in euros per inhabitant of a local municipality from the year 2019 [56]. The municipal share of income tax is 15%, but this is capped at a maximum of 35,000 € for single persons and 70,000 € for married couples assessed together. The remaining tax revenues are due to the federal and state governments [57]. 

Population density and number of residents: The population density was also taken from the Rhineland-Palatinate Statistical Office [56]. The population per square kilometre of the individual municipalities as of 12/31/2021 was recorded [56].

Presence of a hospital: Municipalities with hospitals were identified on the official homepage of the Bad Kreuznach district [58, 59]. Hospitals are located in a total of four municipalities: Bad Kreuznach, Kirn, Bad Sobernheim, and Meisenheim.

Regional population potential: Data on regional population potential was obtained from the online database INKAR (German abbreviation: Indikatoren und Karten zur Raum- und Stadtentwicklung) of the Federal Institute for Research on Building, Urban Affairs and Spatial Development in Germany [60]. The present data are available at the level of the collective municipalities [50] from 2019 onwards.

Regional population potential describes the “potential of inhabitants living within a 100 km radius of a place“ [61]. It is calculated from the sum of the area-weighted municipal population in 1000 within a 100 km air-line distance and is considered a measure of the potential spatial interactions of inhabitants [61].

### Basic data on municipalities

The following basic data on the individual municipalities was taken from the online municipal directory of the joint statistics portal of the federal and state statistical offices (data as of 09/30/2021): municipal association affiliation, area (km^2^), total number of inhabitants, number of female and male inhabitants and age distribution of the individual municipalities (age classes 0–3, 3–6, 6–10, 15–18, 18–20, 20–25, 25–30, 30–35, 35–40, 40–45, 45–50, 50–55, 55–60, 60–65, 65–75 and older than 75 years).

When selecting the variables for this study, we have oriented ourselves towards the variables selected in existing studies, as well as towards the common classification of socioeconomic indicators. We have classified the most commonly used variables into five basic categories: Economic circumstances, housing conditions & infrastructure, mobility & accessibility, education, and household size. Next, we attempted to cover these basic areas as accurately as possible using the available municipality-level data, even if the exact same variables were not available. The small spatial unit, in particular, limited the selection possibilities of the available socioeconomic data.

The revised GISD consists of three subscores: employment, income, and education. Employment data is available at the municipal level, while income data is available at the municipal association level. Education data is only available at the county level. GISD values are normalized on a continuous scale between 0 and 1 for each year. The GISD5 values for 2020 correspond to quintile regions. Bad Kreuznach county is a rural region with GISD5 2020 values of 4 and 5 [55].

We included the GISD employment subscore for 2019 since it is available at a sufficiently small spatial level, but we did not include the other two subscores due to their larger spatial level.

Of the variables we selected, the GISD employment subscore and income tax per municipality represent the economic circumstances, while the population density represents the community infrastructure, and indirectly indicates on housing conditions.

Income tax per inhabitant was used as a proxy for a municipality´s socioeconomic status. Previous research has demonstrated that lower socioeconomic status is often associated with a higher vulnerability to infectious diseases, due to limited opportunities to adhere to preventive measures or less favorable living conditions. Despite capping the municipal share, differences between the individual municipalities can be adequately represented based on differences in wage levels and employment rates. As there is evidence that differences in municipal income tax revenues are directly related to differences in wage levels and employment rates, this dimension reflects socioeconomic disparities adequately [62]. 

Municipalities with a higher population density are more likely to have rather apartments than spacious single-family houses and they tend to have fewer recreational areas for outdoor activities. Regional population potential is evaluated as a parameter for accessibility. However, the Regional Population Potential was only available at the level of collective municipalities. Nevertheless, we included the Regional Population Potential in our evaluation, as it measures the potential for spatial interaction and depicts the accessibility and potential of inhabitants of the various collective municipalities.

No usable measurement variables for the frequently used socioeconomic variables of education and household size were available at the municipal level for the rural district of Bad Kreuznach.

It is important to acknowledge that some critical dimensions, such as employment structure, household size and education level, could not be included due to constraints on the availability of data. Nevertheless, the available variables still offer valuable insights into the local dynamics of the pandemic and pave the way for future research that could incorporate further dimensions.

### Statistical methods and spatial representation

All statistical calculations were performed using the desktop version of RStudio of R Foundation for Statistical Computing (Vienna, Austria) and the “SpatialEpi”, “sf”, “cartography”, “MASS“, and “doBy” packages. Firstly, all the collected data points were merged into a table.

Spatial illustrations use administrative regions from the gadm.org website. Age- and sex-adjusted incidences were calculated using the available numbers on age and sex distribution in the single municipalities from the federal and state statistical offices, with the total age- and sex-distribution numbers for the district of Bad Kreuznach serving as the reference distribution. A search was conducted for potential classes with a significantly higher number of incidences, using the Kulldorff spatial cluster detection method. We use circular clusters which may include up to 50% of the total population, to compare the observed number of cases in each region to the expected number using a poisson model. Significances were estimated using Monte Carlo sampling based on 10,000 samples. The analysis was performed using the Kulldorff function in the SpatialEpi package of R.

First, the influence of individual socioeconomic factors on the municipalities’ cumulative COVID-19 incidences was examined in univariate analyses. Subsequently, a multivariate negative binomial generalized linear model was used to test the influence of these socioeconomic factors on the expected cumulative COVID-19 incidences of the municipalities. As usual, this results in fitting the regression coefficients β_0_, β_1_, …, β_k_ with respect to.

$$\mathrm{E}\left(Y_i\right) = \mathrm{exp} \left(\beta_0 +\beta_1 * \mathrm{X}_{\mathrm{i1}} + ...+\beta_k*\mathrm{X}_{\mathrm{ik}} \right) * \mathrm{E}_\mathrm{j}$$

using *k* different socioeconomic factors *X*_*ik*_ on the counts of COVID-10 incidences *Y*_*i*_ for all municipalities *i* and assuming a negative binomial distribution for the counts with overdispersion parameter θ which is also estimated. This regression formula incorporates the expected number of incidences E_i_ according to the respective age and sex distribution within the single municipalities and the incidences in the whole district as log-offset. As in chi-squared tests, the expected numbers are calculated in the usual way. They focus on socioeconomic factors and adjust for differences in incidence that can be explained by the age and sex distribution in individual municipalities. The regression equation now shows how we include the expected numbers as an offset.

The income tax data was also logarithmized, as is also the case with the GISD income subscore. The values of the socioeconomic factors employment score and income tax score were normalized between 0 and 1, again mimicking the GISD approach. In a further step, these values were pooled across municipal associations and scaling parameters from normalizing the whole data. The socioeconomic factors were pooled across municipal associations to make it more consistent with the GISD, which is only available at the municipal association level.

As the pooled employment subscore was not significant and was highly correlated with the other variables, we removed this factor from our final model. We compared the results of the regression model using the significant factors with a mixed effect model using the municipal associations as grouping variable. The results are highly similar and the random effect is estimated small, therefore, these results are not reported here. In addition, we also calculated the regression model with the numbers of the second wave only. The results are highly comparable showing the same signs in the coefficients and very similar p-values. Again, the results are not reported here.

A comparable approach was used in the sensitivity analysis without the city of Bad Kreuznach.

The significance level was set at 5%.

## Results

A total of 2,881 COVID-19 infections were observed in 103 municipalities. During the observation period, no infections were registered in 15 municipalities. The 158,746 inhabitants in 118 municipalities formed the basis for the collected socioeconomic influencing variables.

### Incidence by age and sex

**Fig. 1:**
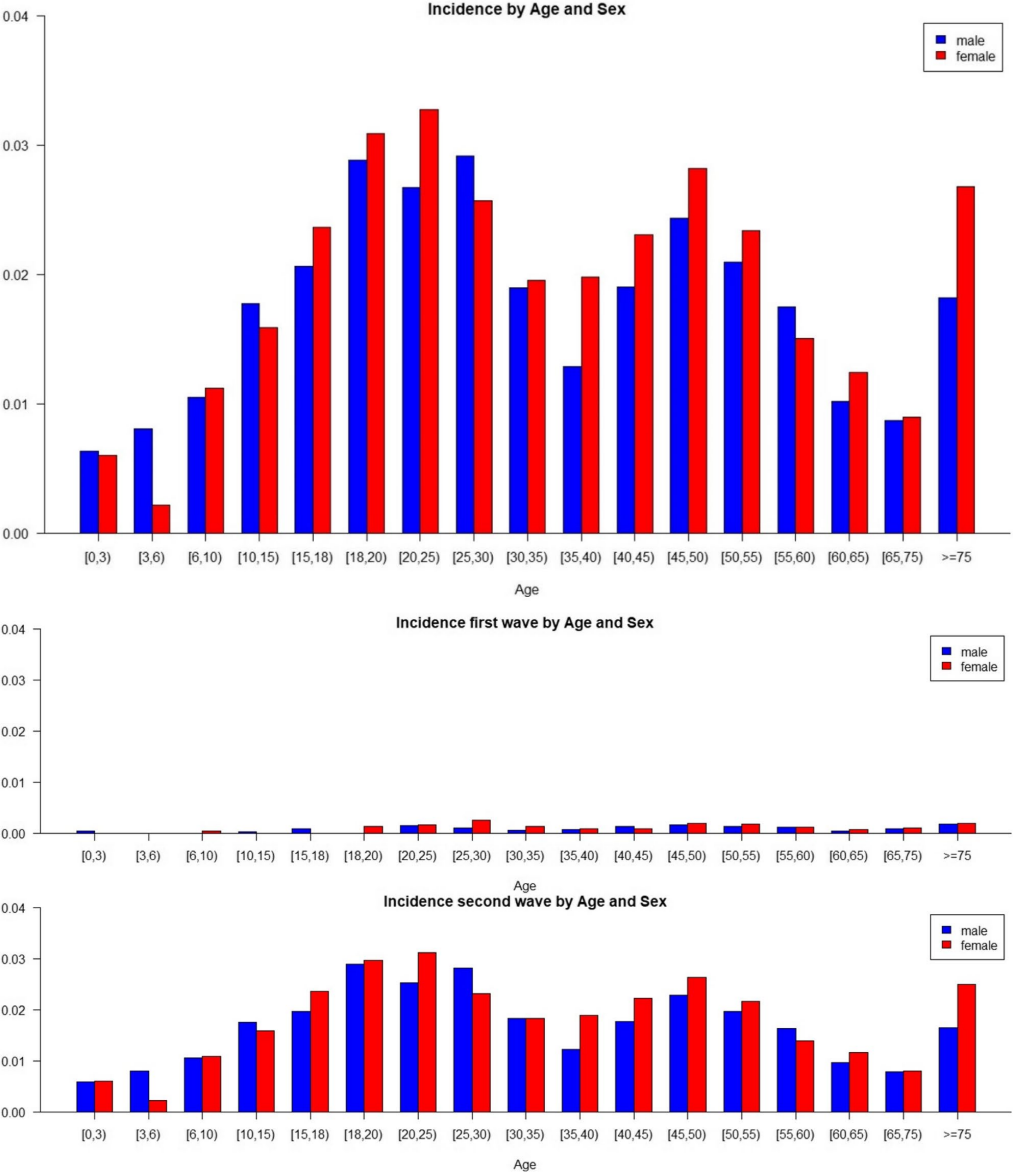
Incidence by age and sex

Figure 1 shows the incidence of Sars-CoV 2 infections by age and sex, pooled over all municipalities in the district of Bad Kreuznach. Data were collected from the first incidence in this region in 03/04/2020 to 01/06/2021, as well as being separated into the first wave of infections (03/04/2020 to 06/30/2020, middle panel) and the second wave of infections (07/01/2020 to 01/06/2021, lower panel).

### Geographical distribution

**Fig. 2:**
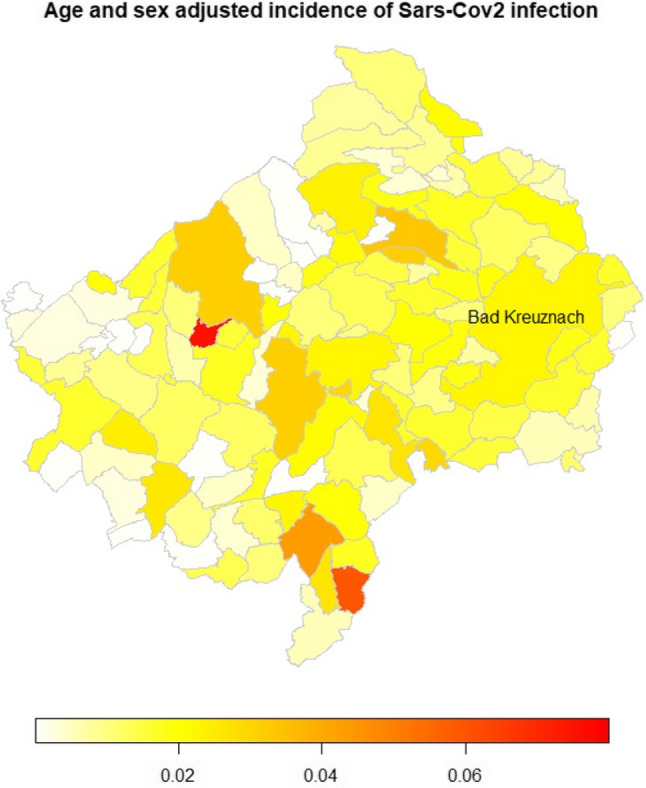
Geographical distribution of age and sex adjusted incidence od Sars-CoV2 infections in the district of Bad Kreuznach

The geographical distribution of SARS-CoV-2 infections in the Bad Kreuznach district during the observation period (Fig. 2).

Although no obvious clusters can be identified, there is considerable heterogeneity between districts. The reference population for the sex and age adjustments was the sex and age distribution of the entire region.

### Incidence curve by GISD classification

**Fig. 3:**
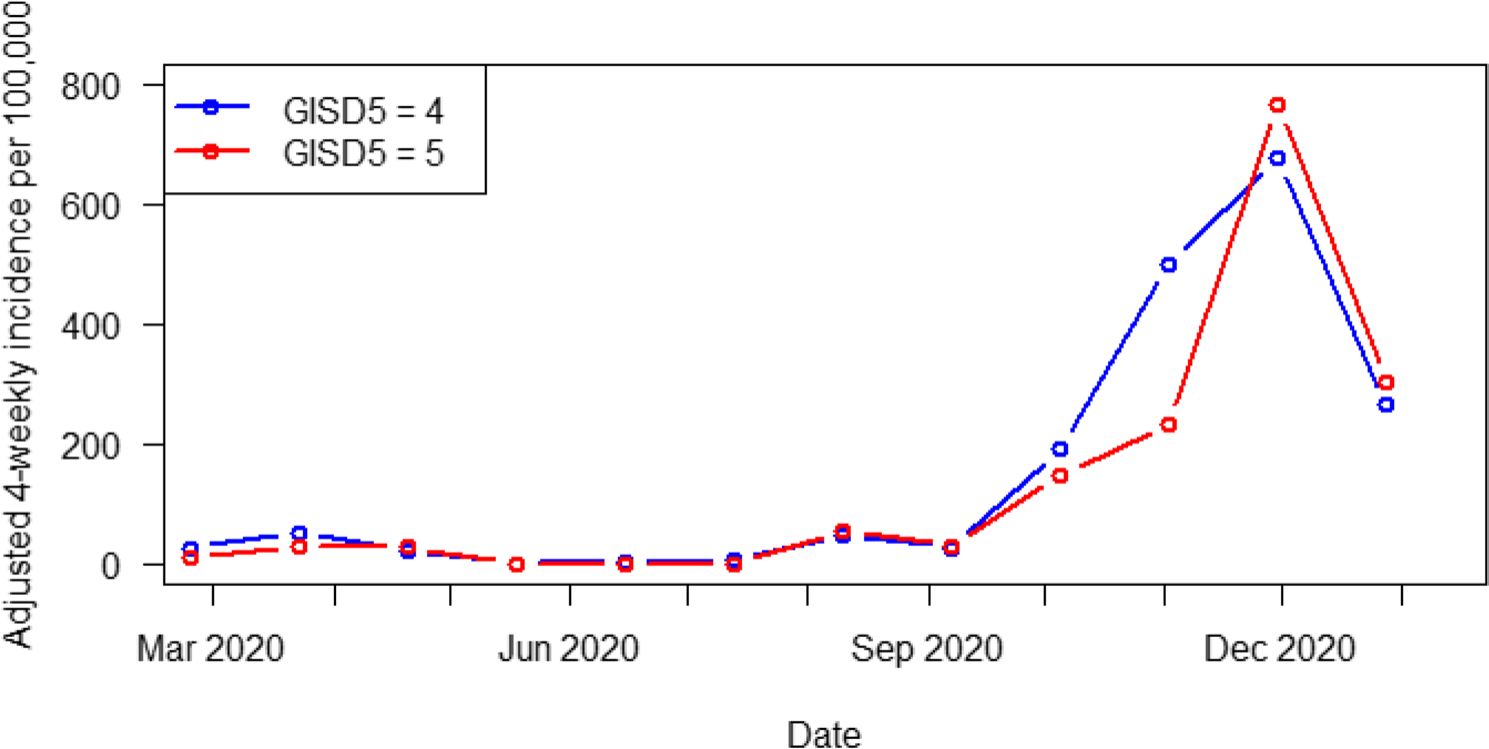
Incidence curve by GISD classification

Figure 3 shows the incidence of COVID-19 infections over time, broken down by GISD classification. It reveals that, during both waves of the pandemic, the incidence rate in less deprived communities (GISD 4, blue) increased more rapidly. During the second wave, the increase in COVID-19 incidence in more deprived communities with a GISD of 5 out of 5 (red) occurred later than that in communities with a GISD of 4 out of 5, but exceeded it.

### Univariate analysis

The graphs of the univariate analyses are presented below. For the GISD employment subscale, consisting of the unemployment and employment rates in 2019, a trend towards higher incidences in more deprived communities (in the district of Bad Kreuznach) was observed. However, this effect was not significant (*p* = 0.80 for the continuous score and *p* = 0.46 for the three-category division).

**Fig. 4:**
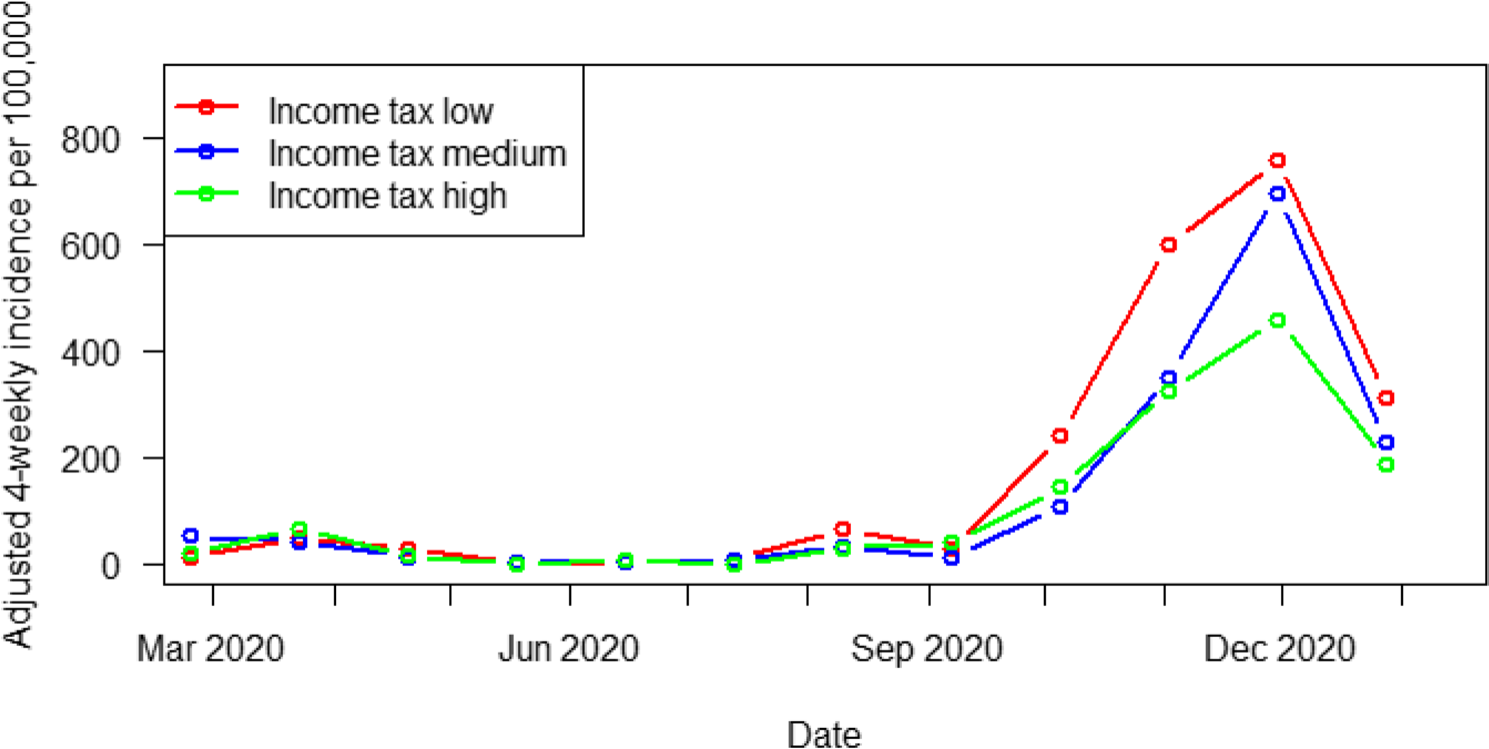
Univariate analysis; income tax

The variable ‘income tax per inhabitant’ was analyzed as raw data for each municipality (on linear and logarithmic scales, as in the GISD), as well as at the level of the municipal association (as in the GISD subscore). The results show that, initially, municipalities with higher income tax (green) were more severely affected at both the municipality level (Fig. 4) and the municipal association level. However, with the second wave of the pandemic, municipalities with lower income tax (red) had higher incidences of COVID-19. No statistically significant results below the significance level were found.

**Fig. 5:**
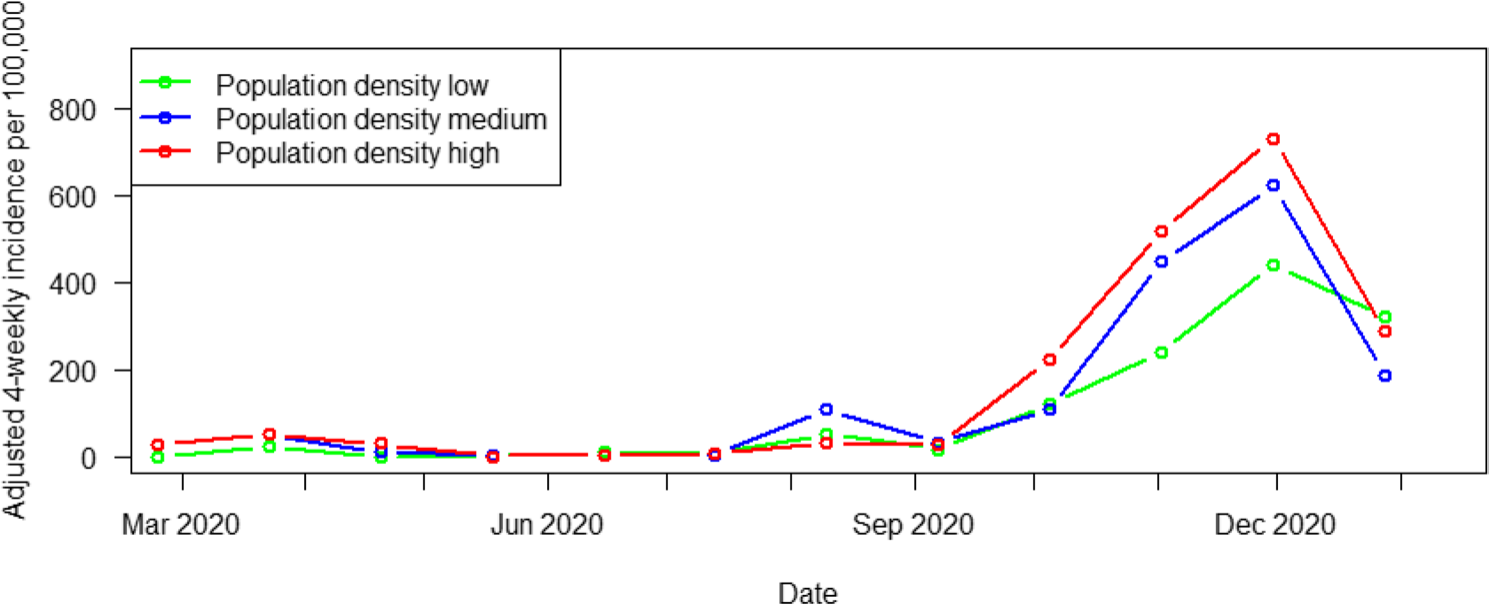
Univariate analysis; population density

Population density was a significant factor when considered as a single continuous variable (*p* = 0.025), with higher COVID-19 incidence rates for municipalities with higher population density (Fig. 5). For the low versus high tertile, *p* = 0.059, and for the low vs. medium tertile, *p* = 0.516. Regional population potential was not significant (*p* = 0.622).

**Fig. 6:**
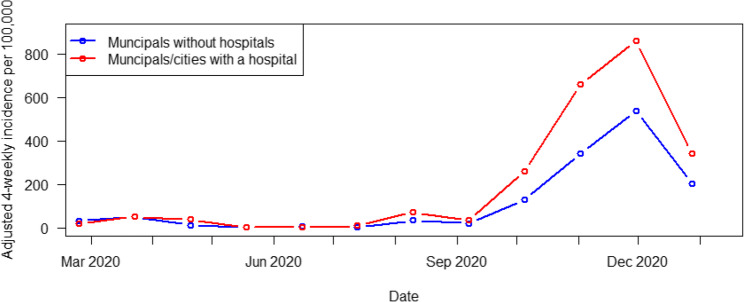
Univariate analysis; hospitals

A very important factor was the distinction between municipalities with and without hospitals (*p* = 0.0009). Municipalities with hospitals had significantly higher COVID-19 incidences than those without hospitals (Fig. 6).

Kulldorff spatial cluster detection

#### Kulldorff spatial cluster detection

**Fig. 7:**
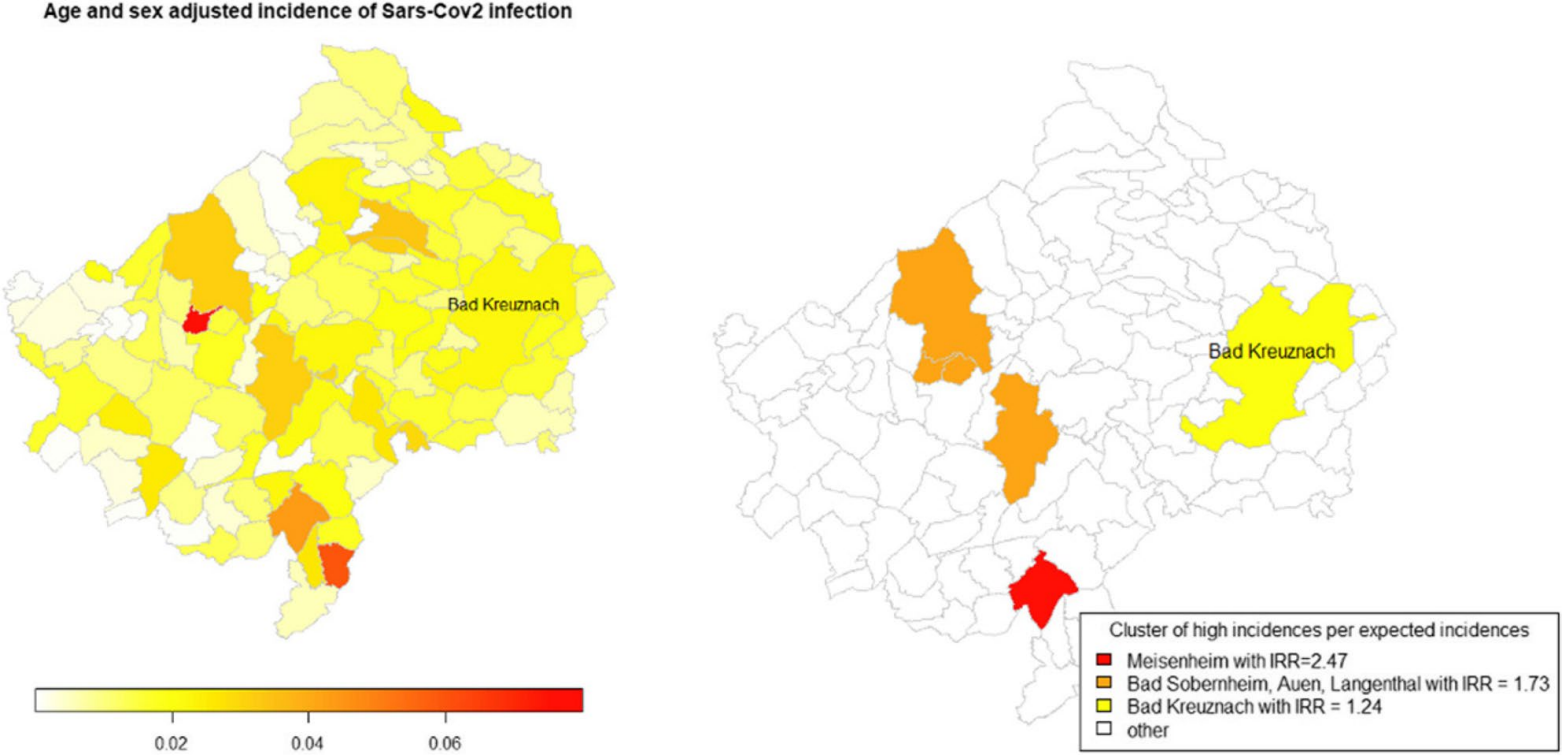
Kulldorff spatial cluster detection

Kulldorff’s spatial cluster detection method was employed to identify communities and potential clusters with high age- and gender-adjusted incidence rates. However, no obvious clusters emerged, as the method is highly sensitive to the threshold value for the proportion of the total population that can be included in each cluster. The cluster estimates are shown with an upper limit of 50%. It should be noted that the orange cluster in Bad Sobernheim comprises of two regions. Panel A of Fig. 7 shows age- and gender-adjusted incidences for comparison purposes, while Panel B on the right shows estimated municipalities and clusters with higher-than-expected incidences.

**Fig. 8:**
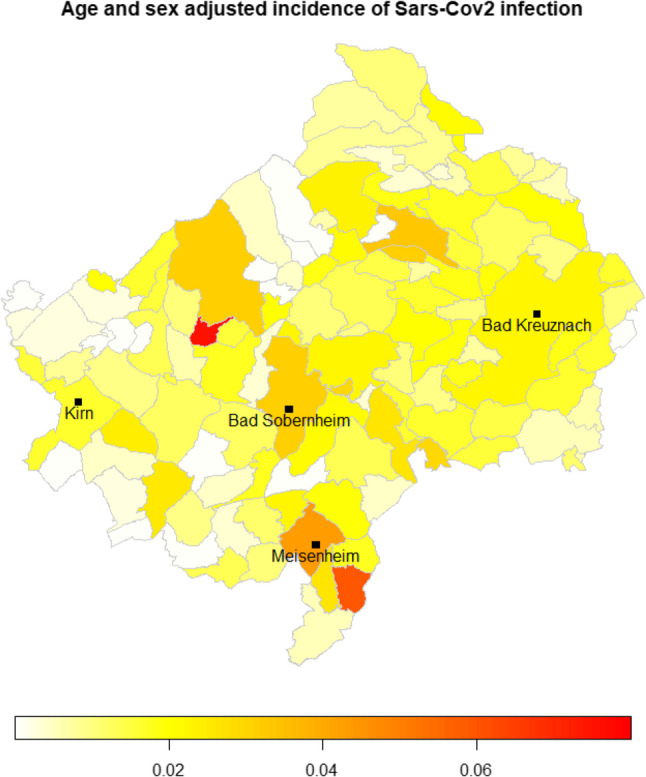
Municipalities with hospitals

For comparison, the municipalities with hospitals are marked in Fig. 8.

### Regression model for socioeconomic factors

Table 1 shows the regression model for socioeconomic factors. This model was used to analyze the potential influence of the selected available socioeconomic factors on COVID-19 incidence.

**Table 1:** Regression model for socioeconomic factors

	Regression coefficient	Standard error	Incidence risk ratio	Confidence interval	*p*-value
GISD employment subscore	-0.625	0.509	0.536	0.192–1.472	0.2197
Income score municipal based	-1.072	0.469	0.342	0.135–0.877	0.0224
Income score collective municipal based	2.914	0.993	18.435	2.721–125.8	0.0033
Regional population potential / 1000	-0.003	0.002	0.997	0.992–1.001	0.1465
hospital	0.773	0.296	2.167	1.267–3.934	0.0091
Inhabitants/1000	0.007	0.014	1.007	0.980–1.037	0.6237
Inhabitants per km²/1000	0.196	0.305	1.217	0.679–2.269	0.5200

We included the GISD 2019 employment subscore and income tax information at the level of collective municipalities, using log transformation and normalisation between 0 and 1 (as with the GISD income subscore). Analogous normalised income tax information at the level of the municipalities themselves, regional population potential, the presence of a hospital, the number of inhabitants, and population density were also included. All of these were incorporated into a multivariable negative binomial regression model with gender- and age-adjusted expected incidences serving as the offset.

The analysis yielded a dispersion parameter fit of 6.1 (SE 1.7), which is significantly greater than 1. Therefore, the negative binomial model appears to be more appropriate than the Poisson model for these count data, for which the dispersion parameter would be equal 1.

The incidence risk ratio describes the extent to which the incidence changes when the influencing factor increases by one unit.

A significant influence was found for income tax at both the municipal (regression coefficient − 1.072; *p* = 0.0224) and municipal association (regression coefficient 2.914; *p* = 0.0033) levels, as well as for the presence of a hospital (regression coefficient 0.773; *p* = 0.0091), on gender- and age-adjusted incidence of COVID-19. No other tested factors had a significant influence on the incidence of the disease with *p* < 0.05. Collinearity analysis yielded a condition number of 38 and variance inflation factors below 2.0 except a variance inflation factor of 2.69 for the number of inhabitants, of 5.9 for the income score on the municipal association level and of 6.1 for the regional population potential. These results suggest some collinearity issues, which appear to be acceptable overall.

### Sensitivity analysis excluding city of Bad Kreuznach

To verify the robustness of the results, a sensitivity analysis was performed in which the city of Bad Kreuznach was excluded. As an independent city, Bad Kreuznach has by far the largest population and population density, and thus differs structurally from the surrounding rural areas. The aim was to verify whether the results remained stable when this potential outlier was excluded.

The analysis confirms the original findings in Table 2. In both analyses, the correlation between income tax per capita and the incidence of cases of the virus remained statistically significant. This confirms that the observed correlation is a statistically robust association. The most significant change was that, while the effect of the presence of a hospital remains comparable to that of the main analysis, the significance level was not maintained. This can be fully explained by the sample size. The other socioeconomic factors show no statistically significant association. We further analyzed the association between the presence of hospitals and the other included parameters, particularly in the sensitivity analysis that excluded the city of Bad Kreuznach. The presence of hospitals was significantly associated with the municipal based income scores and the population size. Including the hospital factor but excluding the income score and the number of inhabitants led to a much more significant association between presence of hospitals and the incidence rates (*p* = 0.000319, detailed analysis data not shown). Therefore, part of the influence of the presence of hospitals may be explained by larger and more prosperous municipals, but this has been adjusted for in the presented analyses.

**Table 2:** Sensitivity analysis without the city of Bad Kreuznach

	Regression coefficient	Incidence risk ratio	Confidence interval	p-value
GISD employment subscore	-0.627	0.534	0.189–1.486	0.2236
Income score municipal based	-1.086	0.338	0.131–0.875	0.0223
Income score collective municipal based	2.890	17.990	2.567–126.6	0.0041
Regional population potential/1000	-0.003	0.997	0.992–1.001	0.1583
hospital	0.705	2.023	1.001–4.330	0.0849
Inhabitants/1000	0.025	1.026	0.905–1.166	0.7033
Inhabitants per km²/1000	0.160	1.173	0.610–2.335	0.6473

## Discussion

In this study, the multivariate regression model revealed a statistically significant association with the expected Sars-CoV 2 incidences for only two factors: income tax per inhabitant and the presence of a hospital. The other factors investigated– GISD employment subscore, inhabitants per km^2^, number of residents, and regional population potential - were found to have no significant association with the expected number of Sars-CoV 2 incidences in the municipalities of the rural district of Bad Kreuznach during the observation period. Our results remained consistent when we excluded the city of Bad Kreuznach from robustness analyses and when we pooled municipalities within potential clusters. As described by the level of rurality in the “Thünen Landatlas”, the district of Bad Kreuznach is a valid example of a rural German district [52].

Looking at the results for income tax, it is notable that the direction of the effect changed between the municipal and municipal association levels. While a negative correlation between income tax per capita and COVID-19 incidence was observed at the municipal level (coefficient − 1.072) – with i.e., higher incomes were associated with lower infection rates – the opposite, positive effect was observed at the municipal association level (coefficient 2.914).

This circumstance can be explained by two possible mechanisms. Firstly, the observed results can be explained by the level of spatial aggregation. At the municipal level, income tax data reflects individual- and household-level socioeconomic conditions. Higher income is generally linked to better living conditions, lower population density, more stable employment, and greater opportunities for working from home and practicing social distancing, which can reduce the risk of infection. At the more aggregated level of the municipal associations, however, the influencing factors are less representative of individual conditions, because aggregation means that local contrasts are less taken into account, and high-income municipalities influence the average. Consequently, high income tax at the municipal association level tends to reflect the structural characteristics of the respective region, such as its economic attractiveness, centrality, job density, and infrastructure. While individual income levels can have a protective effect, these contextual factors can lead to increased infection rates due to greater mobility and contact.

In addition, the change in direction can be partly attributed to statistical mechanisms such as variance reduction and collinearity. When municipalities are grouped together in associations, the heterogeneity within the units decreases, while new correlations with other influencing variables are formed.

A key finding of this study is the significant correlation observed between the presence of a hospital and higher rates of detection of the disease in a community. This association may be explained by two mechanisms: First, hospitals may play a role as mobility hubs for surrounding areas, and may experience higher incidence rates due to centrality and increased mobility. Second, the observed association could also be influenced by differences in testing availability and case detection. Hospitals have not previously been considered in socioeconomic studies. However, other studies have addressed hospitals and other healthcare facilities as potential sources of local infection clusters, as well as the management of nosocomial infections [63, 64].

The present study considers this in the context of socioeconomic conditions and rural areas. As there are only four hospitals in the Bad Kreuznach district and positively reported cases of SARS-CoV-2 were assigned to their respective communities based on their registered addresses, the effect cannot be explained solely by the treatment of patients with SARS-CoV-2 in hospitals. Other factors may also play a role, such as the number of people visiting the hospitals — including patients, medical staff, relatives and external service providers — and the resulting increase in mobility within the municipality, which is the central care provider for the surrounding area. Furthermore, municipalities with hospitals tend to have higher municipal income taxes and larger populations. Even when these factors are considered in a multivariable analysis, it cannot be ruled out that other unobserved socioeconomic characteristics of these municipalities do explain the association.

Nevertheless, this study provides valuable insights and highlights the crucial role of hospitals in tackling the pandemic in rural areas.

In contrast to the findings of this study, other studies have reported significant impacts of various socioeconomic indicators [15–18, 21–27, 65–70]. In Germany, Dragano et al. [24] found that a lower mean household income and less living space per inhabitant were associated with Sars-CoV 2 incidences during the second and third pandemic waves. Further German studies observed the influence of various socioeconomic factors, including poverty among residents, unemployment, lack of vocational training, district income, lack of social capital, environment and safety factors, proportion of the population with a migrant background, unemployment rate, proportion receiving social benefits, average rent, average number of residents per address, and age distribution [25, 27]. These factors were examined in the form of socioeconomic indices used in these studies [25, 27]. Examining the incidences of COVID-19 over time, the studies showed that higher incidence rates were initially observed in the less deprived areas, a trend that was reversed during the second wave of the pandemic [24, 27]. This trend was confirmed in the present study when examining the COVID-19 incidence rates over time according to the GISD classification.

In an international study, a significant association was found between the percentage of unemployed and SARS-CoV-2 2 incidence [66]. Other socioeconomic indicators that were statistically significantly associated with SARS-CoV-2 incidences include the Gini coefficient [67], as well as the percentage of uninsured people, and those living below the poverty line [66].

While these measures are not identical to the variables used in this study, some of them do cover similar socioeconomic areas: Unemployment, the GISD employment subscore, the mean household income and income tax per inhabitant were used as proxies for economic circumstances and poverty [24]. Lower income levels typically signal limited resources, crowded living conditions, and higher dependency on public transport, all of which can contribute to higher transmission rates. In contrast, wealthier areas may benefit from better healthcare infrastructure and access to information, which can reduce infection rates. While the living space per inhabitant is a more direct measurement of living conditions, population density can also be used as a proxy and has already been investigated in the context of Sars-CoV 2 [43]. Densely populated areas, particularly those with more apartment blocks, could increase the likelihood of close contact and transmission.

Due to a lack of available data, factors used in other studies [24, 67], such as employment sectors, educational measures, or the Gini coefficient, could not be used in this study, which affects its comparability. A key distinction between this study and other German investigations [24–27] lies in its geographic and administrative focus. While this study focused on a rural area at the level of individual municipalities, other German investigations often examine metropolitan regions or broader spatial units, such as the Federal Republic of Germany [24], Bavaria [25] or Cologne [27]. These differences in scale and context could contribute to the variability observed in results. In Bavaria [25], an association was shown between Sars-CoV 2 incidence and mortality to an Index of Multiple Deprivation. The index comprised the seven socioeconomic domains: income, employment, education, district revenue, social capital, environment, and security. Furthermore, higher levels of income, employment, education, and social capital were solely associated with reduced Sars-CoV 2 incidence and mortality [25]. In the urban area of Cologne [27], a lower score on a social index consisting of migration background, unemployment, proportion of social benefits, age distribution, average number of residents per address, and the level of rent was associated with higher Sars-CoV 2 incidences. The present study also used the socioeconomic categories income, unemployment, and population density, which are also included in these studies [24–27]. The reduced influence of socioeconomic factors observed in our study may reflect structural differences between rural and metropolitan areas. Various studies [36–38] have described contextual factors unique to rural settings, such as community structures, lower mobility, or differential healthcare access, which may contribute to these deviations. For instance, rural areas may exhibit lower overall mobility and fewer opportunities for social interaction, alongside distinct social interaction patterns and varying access to healthcare resources. These factors could collectively influence the patterns of viral transmission. Further studies using individualized data, mechanistic models or mixed-method approaches are required to validate and expand upon these findings. These contextual differences emphasize the importance of considering local characteristics when analyzing the relationship between socioeconomic factors and infectious disease dynamics, as well as adapting analytical frameworks to account for the distinct dynamics of rural populations.

Another possible explanation is that the influence of socioeconomic factors becomes statistically significant if the number of infections is large enough, regardless of whether urban or rural communities are considered. Smaller spatial units, such as municipalities, may exhibit greater heterogeneity and variability, which could dilute broader trends.

Nevertheless, this would have significant implications for rural areas as well, where small communities prevail.

While our findings contribute to our understanding of local pandemic dynamics, there are several critical limitations and methodological concerns that require detailed discussion in order to contextualize the results and avoid overstating their implications.

The results of the study should be interpreted bearing in mind the observational nature of the data and the statistical approach chosen. While a negative binomial regression model can indicate associations, it does not control unobserved confounding factors or establish causal pathways. For instance, individual-level or district-specific variables such as access to healthcare, personal behavior, or unmeasured socioeconomic disparities could influence the outcomes. Furthermore, the study did not account for overlapping factors such as differences in infection detection methods between municipalities, which could have biased the results. In addition, collinearity analysis still indicates some collinearity problems of moderate severity in the regression analysis, even though the standard errors are still acceptable. These limitations pose significant challenges to the validity of the findings.

A central limitation is that only individuals with a positive PCR test were included, meaning that asymptomatic patients or symptomatic individuals who did not undergo testing were not captured. These untested individuals may be unevenly distributed across different socioeconomic groups, differing in factors such as access to medical facilities, health literacy, education, time constraints, and attitudes towards the pandemic.

The selection of variables was critically constrained by the availability of data at the municipal level. Although variables such as the GISD employment subscore, income tax per inhabitant, population density, and regional population potential were used as proxies for socioeconomic conditions, key dimensions—such as educational attainment and employment sector composition—are missing. These omissions limit the analysis´s comprehensiveness and could mask important explanatory mechanisms. Future studies must include these variables to provide a more nuanced understanding of the socioeconomic determinants of Sars-CoV 2 incidence.

Furthermore, the smaller population and case numbers in rural areas reduce statistical power, making it more difficult to identify significant associations.

These aspects would allow for a deeper exploration of the mechanisms driving the observed associations and enhance the explanatory power of the findings. Overall, rural areas are home to almost 60% of Germany’s total population [52], which highlights the need and importance of knowledge for these regions.

Ultimately, findings at the local level of individual counties and municipalities are crucial for pandemic control. Decisions are made in the individual health departments at this level, and the knowledge gained is ultimately applied.

In light of ongoing geopolitical instability and the priorities of the European Union’s Cohesion Policy for the period 2028–2034, policies for rural areas should prioritize strengthening the resilience of the healthcare sector. For instance, as was evident during the pandemic, this could entail mobile vaccination services, locally tailored information campaigns, and accessible testing services. Here, local findings as presented in this study can play an important role, for example by targeting municipalities with lower income tax for information campaigns and vaccination services. Additionally, targeted preventive measures should incorporate specific strategies for hospitals in rural areas to account for their central role, according to the significant findings in this study. Such policies can reduce disparities between rural and urban regions and increase resilience by improving healthcare monitoring and access.

To strengthen these aspects, further findings for small-scale spatial units are important for detecting infection risks and vulnerable groups, and for understanding the processes behind them. Thus, “think small” instead of “think big” must be employed - insights gained on a large scale must be confirmed on a small scale to provide actual support for decision-making in a pandemic response. By strengthening public health monitoring through the use of fine-grained spatial data, the resilience of rural areas can be enhanced—not only in response to pandemics such as COVID-19, but also to other unexpected external shocks. Therefore, this study contributes to reducing inequalities on two levels: health disparities between population groups and structural disparities between rural and urban areas.

By identifying associations between socioeconomic factors and Sars-CoV 2 incidence, this study provides a foundation for future research to expand and refine our understanding of health inequalities during pandemics. Ultimately, the study reinforces the need for evidence-based, context-sensitive public health policies that address the distinct vulnerabilities of rural communities, thereby contributing to broader efforts to promote health equity and pandemic resilience.

## Conclusion

This study examined the influence of the following socioeconomic factors on Sars-CoV 2 incidence rates (unvaccinated persons) in the municipalities of the rural district of Bad Kreuznach (Rhineland-Palatinate, Germany) from 03/04/2020 to 01/06/2021:GISD employment subscore, income tax per resident (at municipality and municipal association levels), population density, number of residents, presence of a hospital, and regional population potential. A multivariable regression model revealed a statistically significant influence of the variable income tax per capita at both the municipal (*p* = 0.0214) and the municipal association level (*p* = 0.0022). In addition, a significant correlation was found with the presence of a hospital (*p* = 0.0079). No statistically significant influence was found for the other tested socioeconomic factors. Excluding the city of Bad Kreuznach did not change the main results.

These findings differ from those of studies conducted in larger spatial units, which have consistently revealed significant associations with multiple socioeconomic factors. This discrepancy highlights the potential limitations of applying findings from metropolitan or larger-scale studies to local contexts. Consequently, this study offers valuable insights and decision support to help local health departments and policymakers to better address the needs of rural communities during public health crises.

## Data Availability

All data generated or analysed during this study are included in this published article.
